# *HS6ST1* Insufficiency Causes Self-Limited Delayed Puberty in Contrast With Other GnRH Deficiency Genes

**DOI:** 10.1210/jc.2018-00646

**Published:** 2018-06-20

**Authors:** Sasha R Howard, Roberto Oleari, Ariel Poliandri, Vasiliki Chantzara, Alessandro Fantin, Gerard Ruiz-Babot, Louise A Metherell, Claudia P Cabrera, Michael R Barnes, Karoliina Wehkalampi, Leonardo Guasti, Christiana Ruhrberg, Anna Cariboni, Leo Dunkel

**Affiliations:** 1 Centre for Endocrinology, William Harvey Research Institute, Barts and the London School of Medicine and Dentistry, Queen Mary University of London, London, United Kingdom; 2 Department of Pharmacological and Biomolecular Sciences, University of Milan, Milan, Italy; 3 University College London Institute of Ophthalmology, University College London, London, United Kingdom; 4 Centre for Translational Bioinformatics, William Harvey Research Institute, Barts and the London School of Medicine and Dentistry, Queen Mary University of London, London, United Kingdom; 5 NIHR Barts Cardiovascular Biomedical Research Unit, Queen Mary University of London, London, United Kingdom; 6 Children’s Hospital, Helsinki University Hospital and University of Helsinki, Helsinki, Finland

## Abstract

**Context:**

Self-limited delayed puberty (DP) segregates in an autosomal-dominant pattern, but the genetic basis is largely unknown. Although DP is sometimes seen in relatives of patients with hypogonadotropic hypogonadism (HH), mutations in genes known to cause HH that segregate with the trait of familial self-limited DP have not yet been identified.

**Objective:**

To assess the contribution of mutations in genes known to cause HH to the phenotype of self-limited DP.

**Design, Patients, and Setting:**

We performed whole-exome sequencing in 67 probands and 93 relatives from a large cohort of familial self-limited DP, validated the pathogenicity of the identified gene variant *in vitro*, and examined the tissue expression and functional requirement of the mouse homolog *in vivo*.

**Results:**

A potentially pathogenic gene variant segregating with DP was identified in 1 of 28 known HH genes examined. This pathogenic variant occurred in *HS6ST1* in one pedigree and segregated with the trait in the six affected members with heterozygous transmission (*P* = 3.01 × 10^−5^). Biochemical analysis showed that this mutation reduced sulfotransferase activity *in vitro*. *Hs6st1* mRNA was expressed in peripubertal wild-type mouse hypothalamus. GnRH neuron counts were similar in *Hs6st1^+/−^* and *Hs6st1^+/+^* mice, but vaginal opening was delayed in *Hs6st1^+/−^* mice despite normal postnatal growth.

**Conclusions:**

We have linked a deleterious mutation in *HS6ST1* to familial self-limited DP and show that heterozygous *Hs6st1* loss causes DP in mice. In this study, the observed overlap in potentially pathogenic mutations contributing to the phenotypes of self-limited DP and HH was limited to this one gene.

## Teaser

A damaging mutation in *HS6ST1* was found to cause familial self-limited delayed puberty (DP), and heterozygous *Hs6st1* loss produced DP in mice. Mutations in other GnRH deficiency genes were not seen.

Abnormal pubertal timing affects >4% of adolescents and is associated with adverse health and psychosocial outcomes (1–3). However, in most patients with early or late onset of puberty, the underlying pathophysiology is unknown. Self-limited delayed puberty (DP) represents an extreme variant of normal pubertal timing and has been shown to cluster in families (4). Several groups have noted that DP segregates in an autosomal-dominant pattern (4, 5), suggesting monogenic or oligogenic inheritance conferred by haploinsufficiency or a gain-of-function mutation. As such, pedigrees with familial DP represent an invaluable resource to investigate the genetic regulation of puberty onset.

Significant insights into the genetic control of the hypothalamic–pituitary–gonadal (HPG) axis have come from the discovery of rare variants underlying conditions of GnRH deficiency, such as hypogonadotropic hypogonadism (HH) (6). Perturbed embryonic migration of GnRH neurons from the nose to hypothalamus has been shown to cause HH in humans and in animal models (7, 8). Alternatively, HH may arise through mutations that affect GnRH secretion or function in the presence of a normal GnRH neuron number in the hypothalamus (9).

In view of the possible overlap between the pathophysiology of DP and conditions of GnRH deficiency, a few studies have examined the contribution of mutations in genes that cause HH to the pathogenesis of DP (10–12). Potentially pathogenic variants in a small number of genes causing HH (*GNRHR*, *TAC3*, *TACR3*, *IL17RD*, and *SEMA3A*) were identified by whole-exome sequencing (WES) in a few cases of self-limited DP, including those with constitutional delay of growth and puberty (13). Moreover, evidence from WES in our own cohort supports the hypothesis that mutations in genes that influence GnRH neuronal migration or development can cause self-limited DP (14). In this study, we assessed the contribution of mutations in genes known to cause HH to the phenotype of familial self-limited DP and have identified a heterozygous *HS6ST1* mutation as a novel cause of self-limited DP.

## Methods

### Patients

The patients selected for this study belong to a previously described and accurately phenotyped Finnish DP cohort, for which diagnosis is based on objective evidence of a delayed pubertal growth spurt rather than self-recall (15). Patients were referred with DP to specialist pediatric care in central and southern Finland from 1982 to 2004. All patients (n = 492) met the diagnostic criteria for self-limited DP, defined as the onset of Tanner genital stage II (testicular volume >3 mL) >13.5 years in boys or Tanner breast stage II >13.0 years in girls (*i.e.*, 2 SD later than average pubertal development) (16). Medical history, clinical examination, and routine laboratory tests were reviewed to exclude those with chronic illness. HH, if suspected, was excluded by spontaneous pubertal development at follow-up. Normal fertility has been demonstrated in affected family members (5). Families of the DP patients were invited to participate via structured interviews and using archived height measurement records. The criteria for DP in probands’ family members were: (1) age at take-off; or (2) peak height velocity occurring 1.5 SD beyond the mean, that is, age at take-off >12.9 and 11.3 years, or age at peak height velocity >14.8 and 12.8 years in males and females, respectively; or (3) age at attaining adult height >18 or 16 years in males and females, respectively (15). Written informed consent was obtained from all participants. The study protocol was approved by the Ethics Committee for Pediatrics, Adolescent Medicine and Psychiatry, Hospital District of Helsinki and Uusimaa (570/E7/2003). UK ethical approval was granted by the London–Chelsea National Research Ethics Service committee (13/LO/0257). The study was conducted in accordance with the guidelines of the Declaration of Helsinki.

### Genetic analysis

Genetic analysis was performed in 67 probands with DP from those 67 families with the greatest number of affected individuals in our cohort (male, n = 57; female, n = 10, see Supplemental Fig. 1), 58 affected family members (male, n = 36; female, n = 22), and 35 of their unaffected family members (male, n = 13; female, n = 22). WES was performed on DNA extracted from peripheral blood leukocytes of these 160 individuals, using a Nimblegen v.2 or Agilent v.5 platform and Illumina HiSeq 2000 sequencing. The exome sequences were aligned to the University of California Santa Cruz hg19 reference genome. Picard tools and the Genome Analysis Toolkit were used to mark PCR duplicates, realign around indels, recalibrate quality scores, and call variants.

**Figure 1.**
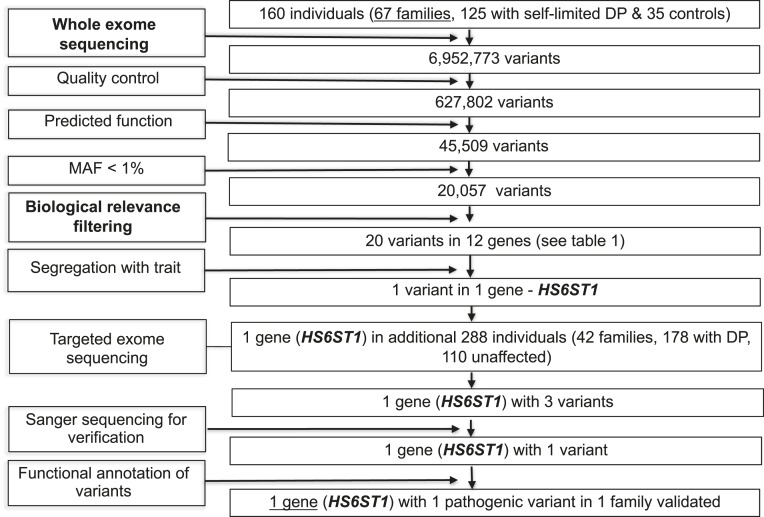
Flowchart of WES filtering strategy to identify *HS6ST1*. WES was performed on DNA extracted from peripheral blood leukocytes of 160 individuals from our cohort (67 DP probands, 58 DP relatives, and 35 controls). The exome sequences were aligned to the University of California Santa Cruz hg19 reference genome. Picard tools and the Genome Analysis Toolkit were used to mark PCR duplicates, realign around indels, recalibrate quality scores, and call variants. Potential causal variants were identified using filters for quality control, predicted functional annotation, MAF, biological relevance (*i.e.*, “HH gene” list), and segregation with trait (see *Methods* and Table 1 for further information on filtering criteria). Targeted exome sequencing using a Fluidigm array of one candidate gene was then performed in a further 42 families from the same cohort (288 individuals, 178 with DP and 110 controls). Variants identified via targeted resequencing were filtered using the same criteria as the WES data, with additional rare variant burden testing. Functional annotation of the variants is as described in “Methods.”

Variants were analyzed and filtered for potential causal variants using filters for quality control, predicted function, minor allele frequency (MAF), and biological relevance (Fig. 1). Filtering by MAF included only variants with MAF <1% in the 1000 Genomes database, the National Heart, Lung, and Blood Institute exome variant server, and the ExAC and gnomAD databases. Biological relevance filtering allowed prioritization of variants according to our “HH genes” list (Table 1), comprising genes known to be relevant to the phenotype of HH based on previously published studies and using pathway analysis with MetaCore (GeneGo/Thomson Reuters). The segregation with trait filter retained only variants present in ≥*n −* 1 affected individuals (where n indicates the number of affected individuals in a given pedigree) and not present in more than one unaffected individual (Table 1).

**Table 1.** HH Gene Filtering

Gene	No. of Rare and Predicated Damaging Variants Filtered From WES Data	
	Not Segregating With the DP Trait	Segregating With the DP Trait
ANOS1	0	0
AXL	3	0
CHD7	3	0
DAX1	0	0
FEZF1	3	0
FGF8	0	0
FGFR1	2	0
GNRH1	0	0
GNRHR	0	0
HESX1	0	0
HS6ST1	1	1
KISS1	0	0
KISS1R	0	0
LEP	0	0
LEPR	0	0
LHX3	1	0
LHX4	1	0
NELF	0	0
PCSK1	0	0
PROK2	0	0
PROKR2	2	0
PROP1	1	0
SEMA3A	0	0
SF1	0	0
SPRY4	1	0
TAC3	1	0
TAC3R	0	0
WDR11	1	0

Twenty-eight HH genes, mutations in which have been identified as causal or potentially causal in patients with GnRH deficiency, used for filtering after WES are shown. Details are given of number of variants in these genes identified in 67 probands with DP after WES. Variants are divided into those that were found to segregate with the DP trait within cohort pedigrees and those that did not segregate.

Targeted exome sequencing (Fluidigm) of the remaining candidate gene postfiltering was performed in a further 42 families from the same cohort (288 individuals, 178 with DP: male, n = 106; female, n = 72; and 110 controls: male, n = 55; female, n = 55; Fig. 1), with filtering as in Howard *et al.* (14). Whole-gene rare variant burden testing was performed after sequencing. A Fisher’s exact test was used to compare the prevalence of deleterious variants in our cohort with the Finnish population, using the ExAC browser (Exome Aggregation Consortium, Cambridge, MA). For each gene, all variants from the ExAC database with minor allele frequency <2.5%, predicted to be deleterious by both Polyphen-2 (17) and SIFT (18), were included in the analysis, with each family in our cohort represented by the proband only. A multiple comparison adjustment was applied *post hoc* using the Benjamini–Hochberg method (19), as detailed in Howard *et al.* (14). All variants identified were confirmed via Sanger sequencing.

### *In vitro* assay of sulfotransferase activity

The human *HS6ST1* cDNA (IMAGE consortium accession ID BC099638) was obtained from GeneCopoeia in a pReceiverM14a backbone (pReceiverM14a-WThHS6ST1). The p.Arg375His and p.Met404Val point mutations were introduced by PCR-mediated mutagenesis (QuickChange II, Agilent Technologies), using the following primers: *Hs6st1*_R375H_FOR, 5′-GCCTGAGGAGCCACGAGGAGCGTCT-3′, *Hs6st1*_R375H_REV 5′-AGACGCTCCTCGTGGCTCCTCAGGC-3′ for p.Arg375His; and *Hs6st1*_M404V_FOR, 5′-CCCACCGAGGACTACGTGAGCCACATCATTG-3′, *Hs6st1*_M404V_REV, 5′-CAATGATGTGGCTCACGTAGTCCTCGGTGGG-3′ for p.Met404Val. Mutations were confirmed by Sanger sequencing. COS7 cells, at 90% confluency in six-well plates, were transfected with 2 μg of wild-type (WT) or mutant plasmids using FuGENE HD (Promega). After 48 hours, cells were lysed with 50 mM Tris-HCl, 150 mM NaCl, 1 mM EDTA, and 1% Triton X-100 (pH 7.4). The homogenates were stirred for 1 hour and then centrifuged at 10,000 × *g* for 30 seconds at 4°C to clear debris. HS6ST1-FLAG fusion proteins were isolated from the supernatant using an anti-FLAG M2 affinity chromatography kit (A2220, Sigma-Aldrich). The purity of the eluted protein was checked by Coomassie brilliant blue (Thermo Fisher) staining of SDS-PAGE electrophoretic gels. Protein relative amounts were calculated by densitometric analysis of Western blots with mouse monoclonal M1 anti-FLAG antibody (1:1000 dilution, Sigma-Aldrich).

Sulfotransferase activity was measured using R&D Systems’ colorimetric assay (EA003). Briefly, different amounts of purified HS6ST1 proteins were added to a mix of 0.2 mM 3′-phosphoadenosine-5′-phosphosulfate (donor), 1 mM *N*-acetylglucosamine (acceptor), and 10 ng/mL inositol monophosphatase 3 (coupling enzyme) in a total volume of 50 μL of reaction buffer. The elution product of mock-transfected cells was used as negative control. The reaction was allowed to proceed for 20 minutes at 37°C with inorganic phosphate measured immediately after by the malachite green method. Enzymatic activity was represented as percentage of WT activity.

### Animals

Animal procedures were performed in accordance with institutional and UK Home Office guidelines. The *Hs6st1^+/−^* mice have been described previously (20, 21) and were maintained on a C57BL/6J background by pairing with WT partners. *Hs6st1^+/+^* and *Hs6st1^+/−^* female mice were checked daily for vaginal opening (VO) and weight after weaning, that is, from 21 days of age (22).

### Histological analyses

Peripubertal and adult mice were perfused with 4% formaldehyde in PBS and then brains and testis were dissected and postfixed overnight at 4°C. Testes were dehydrated, embedded in paraffin, microtome sectioned at 8 μm. and stained with hematoxylin and eosin, as previously described (23). For *in situ* hybridization (ISH) or immunostaining, brains were cryoprotected in 30% sucrose and frozen in OCT compound (VWR). Twelve-micrometer-thick serial sagittal and coronal cryosections were collected on Superfrost Plus slides (VWR) and incubated with antisense riboprobes for *Gnrh* (24) or *Hs6st1* (21). In some experiments, we combined ISH with GnRH immunofluorescence and used a different mouse *Hs6st1* probe, which was generated by PCR amplification from brain cDNA using 5′-ACTGGACCGAACTCACCAAC-3′ and 5′-AACTCAGTGAGGCCGAAGAA-3′ as primers and cloning into the dual promoter vector pGEM-T easy (Promega). Probes were generated as described (25). For immunoperoxidase staining, mouse 25-μm mouse brain cryosections were incubated with Bloxall (Vector Laboratories) to quench endogenous peroxidase activity before incubation with GnRH primary antibody (1:1000 in PBS, ImmunoStar) followed by incubation with biotinylated goat anti-rabbit antibody (1:400 in PBS; Vector Laboratories). Immunostaining was developed with the ABC kit (Vector Laboratories) and diaminobenzidine (Sigma-Aldrich), as described (24).

### Image processing and quantification

Images were acquired either using a Leica DM5500B microscope equipped with a DCF295 camera and DCViewer software (Leica) and then processed with Photoshop CS6 and Illustrator CS6 (Adobe) or acquired on an Axioskop 2 Plus microscope (Zeiss) equipped with a TCH-5.0 ICE digital camera (TiEsseLab). In some experiments, bright-field images of ISH patterns were converted to RGB color mode for superimposition onto fluorescent images. GnRH-positive cells were counted on fifty 25-μm sections through the entire medial preoptic area (MPOA) of each animal. To compare the abundance of GnRH-positive neurites at the median eminence (ME), we measured the pixel intensity of GnRH staining in 25-μm-thick coronal sections through the entire ME.

### Statistical significance

Data are expressed as mean ± SEM. Differences between groups were evaluated by a two-tailed student *t* test and considered significant at *P* < 0.05. Statistical analysis was performed using Prism (GraphPad Software).

## Results

### Exome sequencing of families with self-limited DP identified an *HS6ST1* variant

WES of 67 informative families from our large cohort with self-limited DP identified 20 rare (MAF <1%) and predicted deleterious variants in 12 genes from a list of known HH genes (Table 1). However, after filtering for segregation with trait, only *HS6ST1* (ENSG00000136720, gene ID number 9394) was retained as a candidate gene (Fig. 1). One proband and his affected relatives from one pedigree sequenced carried a rare and likely damaging *HS6ST1* variant [NM_004807.2: c.1124G>A (rs182882999) p.Arg375His] that causes a nonconservative amino acid substitution in the coding sequence. This proband carried an additional potentially pathogenic variant in a known HH gene, *FEZF1* (NM_ NM_001024613.3: c.1010T>A, p.Iso337Lys). However, this variant was not found in any other affected individual from this family and was, therefore, discounted due to lack of familial segregation.

The rare heterozygous missense variant *HS6ST1* (p.Arg375His) is predicted to be deleterious to protein function with five of six prediction tools, as the affected amino acid residue resides in a coiled-coil domain that is highly conserved among species, as revealed by the PhyloP and GERP score and a multiple sequence alignment (Supplemental Fig. 2 and Supplemental Table 1). This specific variant was present at low MAF in some public databases, but whole-gene rare variant burden testing showed significant enrichment of *HS6ST1* rare predicted pathogenic variants in our cohort as compared with ethnically matched controls from the ExAC database (adjusted *P* = 3.01 × 10^−5^).

After targeted exome sequencing, two additional variants in *HS6ST1* were identified in three further affected probands [NM_004807.2: c199A>T (rs202247387) p.Lys67* and c585G>A p.Trp195*]. However, Sanger sequencing did not validate the existence of these variants. No further rare, potentially damaging, and trait-segregating variants in *HS6ST1* were identified by targeted sequencing.

### Pedigree with a potentially pathogenic *HS6ST1* variant displayed an autosomal-dominant inheritance pattern and classical self-limited DP

**Figure 2.**
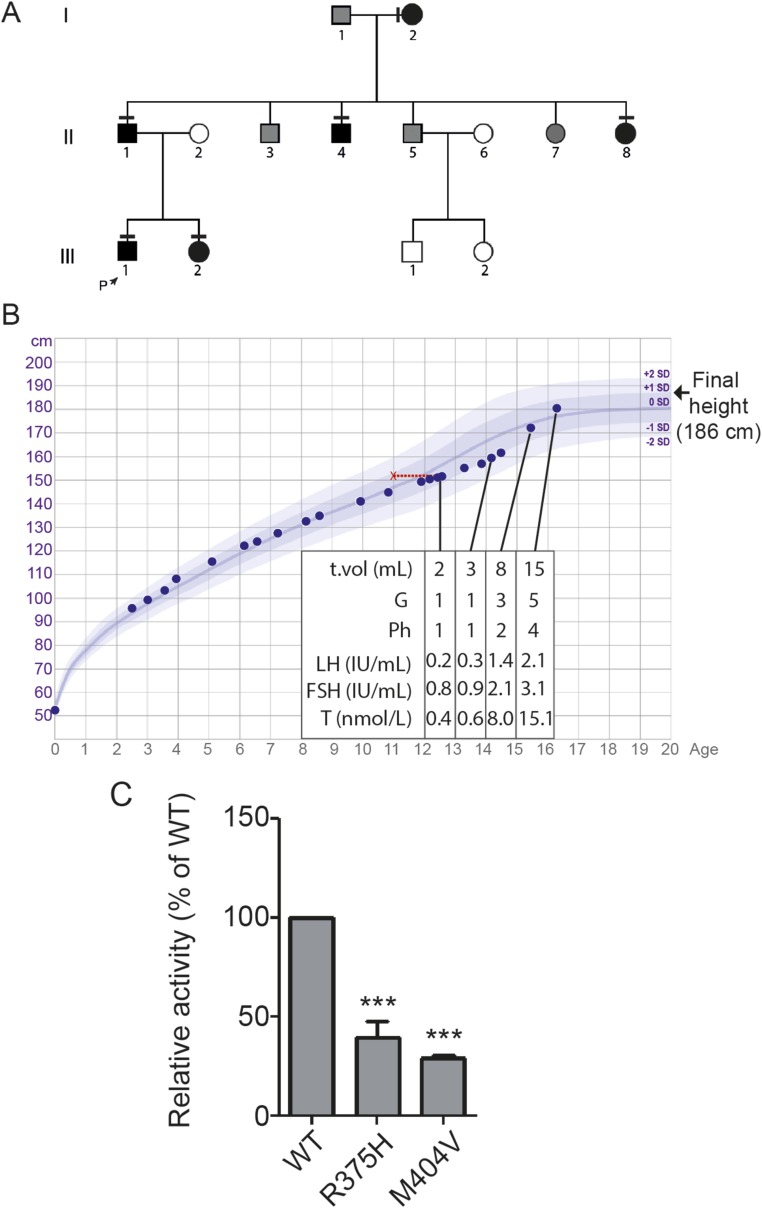
Pedigree and clinical details of a patient with a p.Arg375His mutation in *HS6ST1* that impairs sulfotransferase activity. (A) Pedigree of the proband with the p.Arg375His mutation. Squares indicate male family members; circles indicate female family members. Black symbols represent clinically affected family members, gray symbols represent family members with unknown phenotype, and clear symbols represent unaffected individuals. The arrow labeled “P” indicates the proband in the family. A horizontal black line above or adjacent to an individual’s symbol indicates that they are heterozygous for the p.Arg375His mutation as identified by either WES or Fluidigm array and verified by Sanger sequencing. (B) Clinical details of the proband with the p.Arg375His mutation. Height chart for the proband shows reduction in growth velocity from 12 y of age, with associated delayed bone age shown in red. The subject was prepubertal until 14.1 y of age. This was followed by spontaneous onset of puberty and subsequent development during 2 y to normal adult testosterone levels and testicular volume. (C) Sulfotransferase activity assay. The p.Arg375His mutation reduces *HS6ST1* sulfotransferase activity. Relative specific activity (mean ± SD) of recombinant WT or mutant (p.Arg375His or p.Met404Val) HS6ST1 proteins are shown. All assays were done as three independent biological repeats (n = 3 for each experiment) using equal amounts of protein. ***P < 0.001. G, Tanner genital stage, Ph, Tanner pubic hair stage, T, testosterone; T vol, testicular volume.

The pedigree with the p.Arg375His variant has several family members with typical features of self-limited DP (Fig. 2A). The proband was first investigated for growth delay at 12.8 years, at which time his bone age was 11 years (Fig. 2B). Examination at this stage found him to be prepubertal, with bilateral testis volumes of 2 mL and no pubic hair development. His blood biochemistry at that time showed a typical picture of functional HH. Spontaneous onset of puberty was observed at ∼14.3 years of age. During the next 2.4 years, he achieved testis volume and testosterone levels within the normal adult range. His sister’s age at menarche was 15 years. Both siblings had normal birth weight and birth length. Their father and paternal uncle and aunt also had DP with delayed pubertal growth spurt. All family members with DP had self-reported normal olfaction.

### The HS6ST1 p.Arg375His mutant protein has reduced sulfotransferase activity *in vitro*

*In vitro* analysis of sulfotransferase function of the p.Arg375His mutant HS6ST1 protein (Fig. 2C) showed reduced activity at 39% of the WT HS6ST1 protein (mean ± SD: 39.81 ± 8.21, *P* < 0.001). This reduction in enzymatic function is within the range seen with other pathogenic mutations in *HS6ST1* found in patients with HH (26) and similar to that of the p.Met404Val mutant HS6ST1 protein (mean ± SD: 28.8 ± 1.46). This observation is consistent with the *HS6ST1* p.Arg375His mutation as causative in self-limited DP.

### *Hs6st1* is expressed within the adult hypothalamus and olfactory bulbs

**Figure 3.**
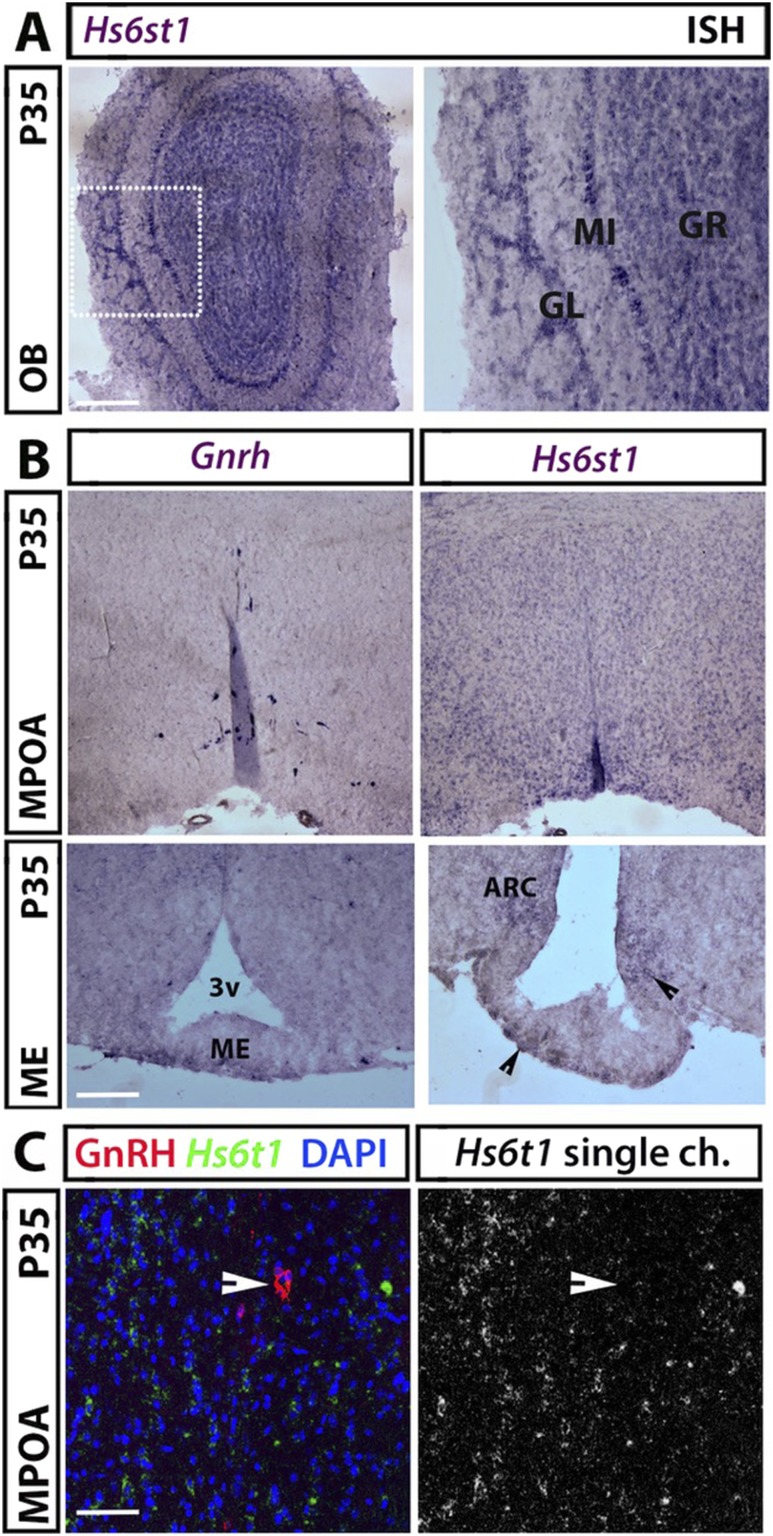
*Hs6st1* mRNA expression in GnRH neuronal territories on P35. (A) Coronal sections of adult mouse OB were labeled for *Hs6st1* by ISH. The squared box in the left image is shown at higher magnification to the right. (B) Contiguous coronal sections of adult mouse hypothalamus representing the MPOA (upper panels) and the ME (lower panels) were labeled for *Gnrh* and *Hs6st1* by ISH; arrowheads indicate the *Hs6st1* expression in the ARC and ME. (C) *Hs6st1* ISH (green) of coronal sections from P35 MPOA followed by immunolabeling for GnRH (red) revealed no expression of *Hs6st1* in GnRH-positive neurons (example of GnRH neuron is indicated with a solid arrowhead). Scale bars: 125 µm (A and B, upper panels), 50 µm (B, lower panels), 25 µm (C). 3v, third ventricle; GL, glomerular layer; GR, granular layer; MI, mitral layer.

In the adult brain, GnRH neurons are scattered in a bilateral continuum between the olfactory bulb (OB) and the MPOA of the hypothalamus, where most cell bodies reside (27). We found that *Hs6st1* is highly expressed in the granular, mitral, and glomerular layers of the adult OB (Fig. 3A) and diffusely in the MPOA (Fig. 3B, upper panel). *Hs6st1* expression was also observed in the arcuate nucleus (ARC), which harbors neurons that regulate GnRH secretion (Fig. 3B, lower panel). However, *Hs6st1* mRNA did not localize to GnRH^+^ neurons (Fig. 3C). These observations suggest that *Hs6st1* is expressed by hypothalamic cells that do not correspond to GnRH neurons.

### Normal positioning and number of GnRH neurons in the MPOA of Hs6st1 heterozygous mice

**Figure 4.**
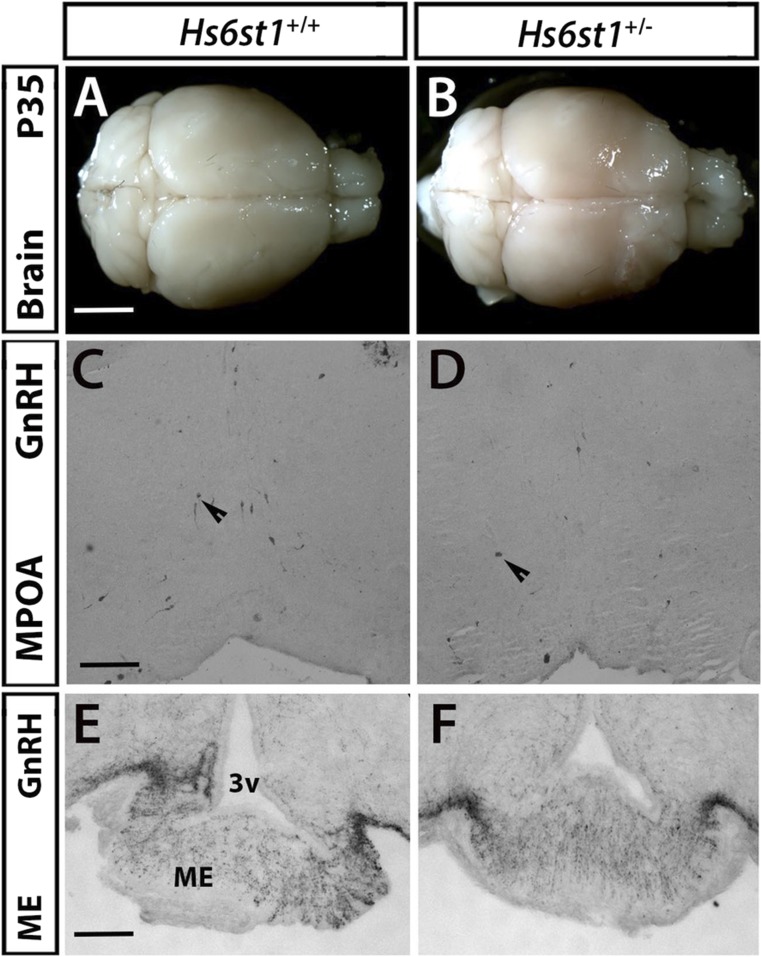
*Hs6st1^+/−^* mice showed no defects in olfactory bulbs or in the number and projections of GnRH neurons. (A and B) Perfused adult *Hs6st1^+/+^* and *Hs6st1^+/−^* brains were dissected, photographed, and showed similar olfactory bulb structure in both genotypes. (C and D) Coronal sections of adult mouse MPOA were immunostained for GnRH; arrowheads indicate examples of GnRH neurons that are normally present both in *Hs6st1^+/−^* and WT mice. (E and F) Immunostaining for GnRH in coronal sections of adult mouse ME shows that GnRH neurons project similarly to the ME in *Hs6st1^+/+^* and *Hs6st1^+/−^* mice. Scale bars: 3 mm (A and B), 125 µm (C and D), 50 µm (E and F). Arrows demonstrate representative GnRH cell bodies. 3v, third ventricle.

To determine whether HS6ST1 is required for the normal development of the GnRH neuron system, we compared mice heterozygous for a *Hs6st1* null allele (*Hs6st1^+/−^*) to their WT littermates (*Hs6st1^+/+^*). However, there were no gross differences in brain or OB anatomy between the genotypes (Fig. 4A and 4B). Moreover, there were no obvious differences in the positioning and number of GnRH neurons in the MPOA (Fig. 4C and 4D; number of GnRH^+^ cells: *Hs6st1^+/−^* 341.2 ± 24.2 vs *Hs6st1^+/+^* 378.3 ± 30.2, n = 5 each; *P* = 0.37). GnRH neurons in *Hs6st1^+/−^* mice also projected similarly to the ME, the site where they normally release GnRH into the portal blood vessels of the pituitary gland; moreover, the pixel intensity of GnRH-stained neurites was similar in both genotypes (Fig. 4E and 4F; percentage stained neurite area: *Hs6st1^+/+^* 2.9 ± 0.4 vs *Hs6st1****^+/−^*** 3.1 ± 0.3, n = 5 each; *P* = 0.09).

### *Hs6st1^+/−^* mice show significant DP onset

**Figure 5.**
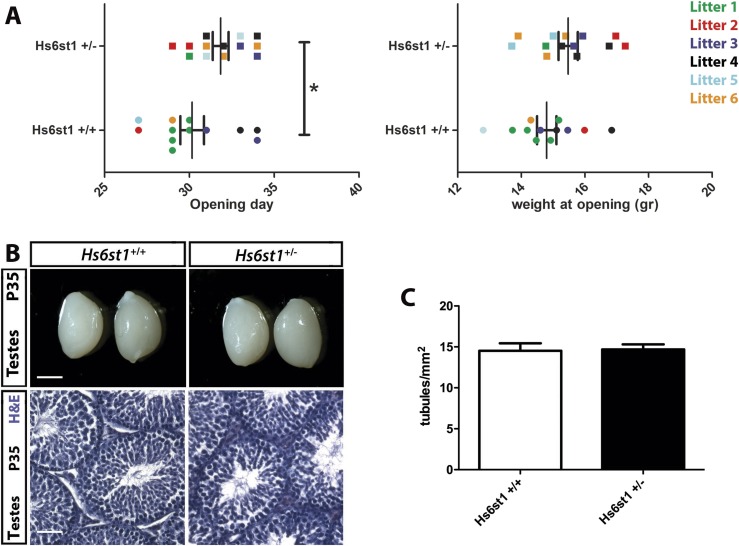
Peripubertal female *Hs6st1^+/−^* mice show delayed VO whereas young adult male *Hs6st1^+/−^* mice showed normal testes morphology. (A) Age (left graph) and weight (right graph) at the time of the VO in female *Hs6st1^+/+^* (n = 12) and *Hs6st1^+/−^* (n = 13) mice. Values for littermates are shown in the same color. *P < 0.05. (B and C) *Hs6st1^+/+^* and *Hs6st1^+/−^* mice have similar testes size (B, upper panels), normal spermatogenesis after H&E staining of paraffin sections (B, lower panels), and similar number of seminiferous tubules (C). Scale bars: 3 mm (B, upper panels), 25 µm (B, lower panels).

To determine whether heterozygous *HS6ST1* deficiency is sufficient to cause DP, we compared the timing of puberty in *Hs6st1****^+/−^*** and *Hs6st1^+/+^* female mice by identifying the day of VO, a proxy measurement for pubertal activation of the HPG axis in mice (22). We found that VO was delayed by on average 1.88 ± 0.82 days in *Hs6st1^+/−^* compared with *Hs6st1^+/+^* females (Fig. 5A, left; postnatal day (P) of VO: *Hs6st1^+/+^* 30.2 ± 0.7, n = 12, vs *Hs6st1^+/−^* 31.9 ± 0.5, n = 13; *P* = 0.04; mice were pooled form six different litters). DP in *Hs6st1^+/−^* females was not due to overall delayed development, as they were not smaller than *Hs6st1^+/+^* littermate females around the time of VO (weight at P30: *Hs6st1^+/+^* 14.12 ± 0.58 g, n = 12, vs *Hs6st1^+/−^*13.67 ± 0.58 g, n = 13; *P* = 0.29). Instead, and consistent with their DP and thus older age at VO in the *Hs6st1^+/−^* females, there was an insignificant trend toward greater weight at the time of VO in *Hs6st1^+/−^* compared with *Hs6st1^+/+^* females within the cohort examined (Fig. 5A, right; body weight on the day of VO: *Hs6st1^+/+^* 14.9 ± 0.3 g, n = 12, vs *Hs6st1^+/−^* 15.5 ± 0.3 g, n = 13; *P* = 0.06).

Despite DP, the fertility of young adult *Hs6st1^+/−^* males and females of both sexes appeared normal, because they produced litters without obvious delay and of normal litter size when paired to WT mice at 4 to 6 months of age (litter size: *Hs6st1^+/−^* × *Hs6st1^+/+^*: 7.2 ± 0.95 pups per litter in six litters, vs *Hs6st1^+/+^* × *Hs6st1^+/+^* 7.3 ± 0.71 pups per litter in seven litters; *P* = 0.89; *Hs6st1^+/−^* mice were born at the expected Mendelian ratio). In agreement with the ability to father litters of normal size, testes size was similar in young adult *Hs6st1^+/−^* and *Hs6st1^+/+^* males (Fig. 5B; P35 testes length (in mm): *Hs6st1^+/−^* 8.30 ± 0.16, n = 6, vs *Hs6st1^+/+^* 8.31 ± 0.17, n = 8; *P* = 0.97). Moreover, testes showed normal organization of germ cells and interstitial Leydig cells and contained a similar number of seminiferous tubules per area (Fig. 5B and 5C; number of seminiferous tubules per mm^2^: *Hs6st1****^+/+^*** 14.53 ± 0.91 vs *Hs6st1****^+/−^*** 14.69 ± 0.62; n = 5 each; *P* = 0.88). Taken together, these findings show that HS6ST1 haploinsufficiency delays puberty in mice without compromising fertility in the adult.

## Discussion

The inheritance of self-limited DP is under strong genetic influence, with clear autosomal-dominant segregation in many families with or without complete penetrance (4), and thus represents a useful basis for the investigation of puberty genetics (5). However, only a few genes responsible for self-limited DP have been identified. In view of the possible overlap between the pathophysiology of DP and HH, screening self-limited DP patients for mutations in known HH genes appears a prudent strategy; however, minimal genetic overlap between the two conditions has been identified to date (13). Additionally, there have been some observed differences in inheritance patterns and penetrance between the two conditions (28). In HH, >40 different genes have been identified, and incomplete penetrance is often seen within pedigrees, leading to a wide spectrum of phenotypes (29–31). As the understanding of the genetic basis of both self-limited DP and HH improves, it is likely that genetic testing will be able to help establish a definitive diagnosis in adolescent patients presenting with delayed onset of puberty.

In this study, we have identified a deleterious mutation, p.Arg375His, in the HH gene *HS6ST1* as the likely causal factor for self-limited DP in one pedigree from our cohort of patients with familial DP.

*HS6ST1* mutations have been previously identified in up to 2% of patients with idiopathic HH (26, 32), but they have not previously been reported in pedigrees segregating with the trait of self-limited DP. Our *in vitro* analysis showed that the *HS6ST1* p.Arg375His mutation had reduced sulfotransferase activity, comparable to previously published mutations in this gene in patients with HH (26). Taken together, these results suggest a working model in which *HS6ST1* heterozygosity causes self-limited DP, whereas potentially *HS6ST1* homozygosity or a “second hit” in a separate gene would produce the phenotype of HH.

Our studies in a murine model corroborate heterozygous *Hs6st1* deficiency as a cause of delayed pubertal timing without compromised fertility. Thus, *Hs6st1*^+/−^ mice were born at normal Mendelian ratios without obvious defects in GnRH neuron or testes development, but females showed delayed VO. The question of sex bias in self-limited DP is an interesting one, as it is diagnosed in up to 83% of boys and 30% of girls presenting with pubertal delay (33–35). The underlying reasons for this sex difference are not clear. However, this difference may be due, in part, to ascertainment bias. Indeed, a previous study has shown a nearly equal sex ratio consistent with autosomal-dominant inheritance when all of the affected individuals from our large cohort were examined, as opposed to the probands only (5).

In our murine model of *Hs6st1^+/−^* mice, we did not find any evidence of a sex difference in anatomical studies. In this study only females were examined for timing of pubertal onset (VO), as VO is a very robust marker of pubertal onset in female mice. Male mice were not assessed for the timing of puberty, but the adult male mice had normal testicular morphology, including the presence of spermatogenesis. Normal spermatogenesis is an excellent marker in males for reproductive competence and excludes GnRH deficiency. In female mice the reproductive competence was assessed as fertility of the females. With these methods GnRH deficiency was excluded both in male and female *Hs6st1^+/−^* mice. Importantly, mice deficient in *Hs6st1* were not significantly smaller or lighter than their WT littermates, excluding poor growth as the underlying cause for pubertal delay. The viability of *Hs6st1*^+/−^ mice is in contrast to that of *Hs6st1* knockout (*Hs6st1*^−/−^) mice, which could not be examined for puberty defects due to embryonic lethality (36).

Our expression studies in mice indicate that *Hs6st1* is expressed in several tissues relevant to normal GnRH neuron migration or function, including the OB and the MPOA, ARC, and PVN of the hypothalamus. However, we did not identify any obvious abnormalities in OB morphology, GnRH neuron number in the MPOA, or in GnRH neuron innervation of the ME in *Hs6st1^+/−^* mice. Instead, *Hs6st1* expression in the ARC and PVN, where kisspeptin neurons and tanycytes modulate GnRH secretion and function (37, 38), raises the possibility that HS6ST1 haploinsufficiency affects the regulation of GnRH neuron activity or other relevant downstream pathways.

*Hs6st1* is required for the function of *Anos1* (also known as *Kal1*) and *Fgfr1*, two genes that are required for normal HPG axis function (26). In a *Caenorhabditis elegans* model, *Hs6st1* regulates neural branching in concert with *Anos1* and *Fgfr1* (26). Moreover, *Anos1* is also able to enhance *Fgfr1* signaling in an HS-dependent manner in an immortalized human cell model of GnRH neurons, and it has been proposed that this interaction promotes olfactory and GnRH neuron development (39). However, *Hs6st1* mutations may also impact the HPG axis via *Anos1*-independent pathways, as HS modifications of many different proteoglycans in the extracellular matrix have the potential to affect multiple signaling pathways (40) previously implicated in the neuroendocrine control of fertility (41).

In summary, we have identified a new pathogenic mutation in *HS6ST1* as the likely cause of DP in a pedigree from our extensive patient cohort, with no other pathogenic mutations in genes known to cause HH identified in our cohort. These findings suggest that, with the exception of a few genes like *HS6ST1,* the genetic background of HH and DP is either largely different or is due to mutations in as yet undiscovered genes. Our findings differ from those in previous publications, in which up to 14% of DP probands were reported to carry potentially pathogenic variants in HH genes (13). However, previous results have not been adjusted for segregation with the DP trait within families, nor have the variants identified in these previous studies been tested for pathogenicity, and thus prior estimates of HH mutation rates in DP patients may have been an overestimation. In agreement, prior to adjustment for segregation, our results suggested that 17.9% of DP probands had potentially pathogenic variants in HH genes, highlighting the value of familial data in identification of causal variants in a condition such as DP. Although further pedigrees with familial DP need to be studied to identify additional causative genes and provide accurate estimates of the genetic HH and DP overlap, our findings provide evidence that perturbations in a single allele of a gene regulating the HPG axis is sufficient to cause self-limited DP. In contrast, available evidence suggests that more deleterious alterations in the same gene, or in combination with additional genes, are required to cause more severe HH phenotypes.

## Supplementary Material

Supplemental Data

## Abbreviations:

ARC: arcuate nucleus
DP: delayed puberty
HH: hypogonadotropic hypogonadism
HPG: hypothalamic–pituitary–gonadal
ISH: *in situ* hybridization
MAF: minor allele frequency
ME: median eminence
MPOA: medial preoptic area
OB: olfactory bulb
P: postnatal day
VO: vaginal opening
WES: whole-exome sequencing
WT: wild-type
