# Supplementary material for: HS6ST1 Insufficiency Causes Self-Limited Delayed Puberty in Contrast With Other GnRH Deficiency Genes
Source: J Clin Endocrinol Metab. 2018 Jun 20;103(9):3420–9. doi: 10.1210/jc.2018-00646 (PMC6126894; doi:10.1210/jc.2018-00646)
Supplement: Supplemental Data [file jc.2018-00646.sd1.pdf]

# **Supplementary Appendix**

*HS6ST1* insufficiency causes self-limited delayed puberty in contrast with other GnRH deficiency genes

Authors - Howard SR, Oleari R, Poliandri A, Chantzara V, Fantin A, Ruiz-Babot G, Metherell LA, Cabrera CP, Barnes MR, Wehkalampi K, Guasti L, Ruhrberg C, Cariboni A, Dunkel L

| AA Change | dbSNP137 | PhyloP[1] Pred | SIFT[2] Pred | Polyphen2[3] Pred | LRT[4] Pred | MutationTaster[5] Pred | FATHMM[6] Pred | CADD[7] Pred | GERP[8]++ |
|---|---|---|---|---|---|---|---|---|---|
| **p.Arg375His** | rs182882999 | C | T | B | D | D | D | D | 4.3 |

**Supplementary Table 1 – Prediction of variants according to web-based prediction software programs and conservation across species.** C – conserved, D – deleterious, disease causing or damaging, B – benign, T - tolerated

| Nucleotide change | Amino acid change | Exon | MAF from DP probands (%) (n=109) | MAF from cohort controls (%) (n=145) | MAF from Exac database (%) Finnish/European/All |
|---|---|---|---|---|---|
| **c.1124G>A** | p.Arg375His | 2 | 0.92 | 0 | 0.03/0.003/0.03 |

**Supplementary Table 2 – Minor allele frequency in study population and control cohorts.** Minor allele frequency data for the control population was retrieved from the ExAC Browser (Exome Aggregation Consortium (ExAC), Cambridge, MA: http://exac.broadinstitute.org, accessed May 2017).

1

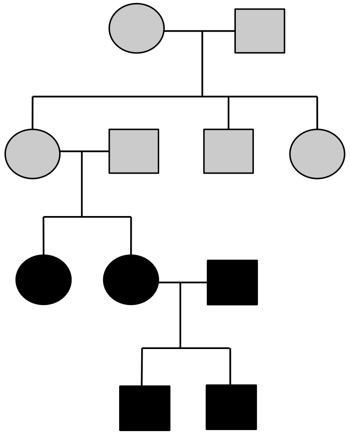

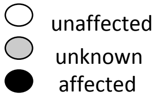


2

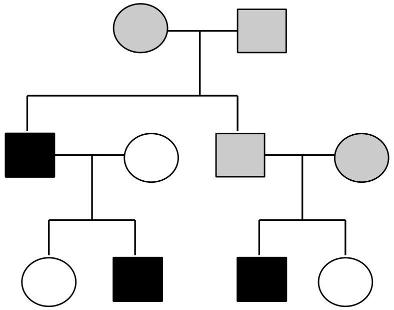

3

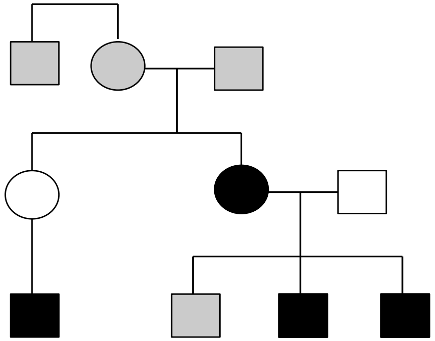

4

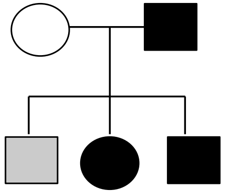

5

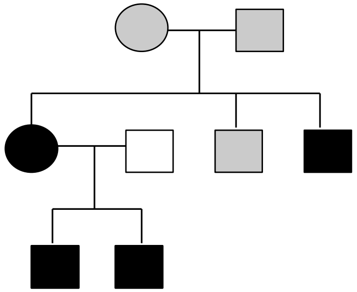

6

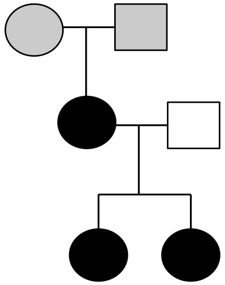

7

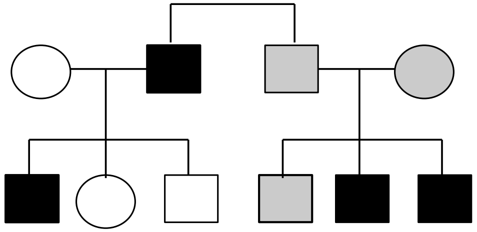

8

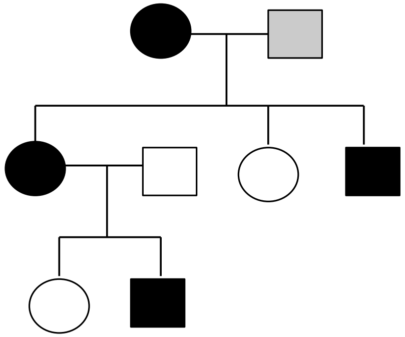

9

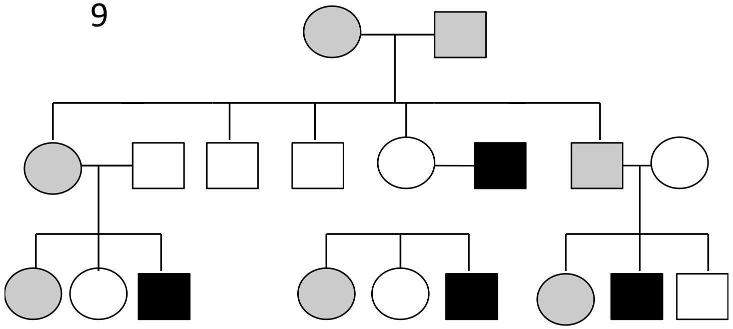

10

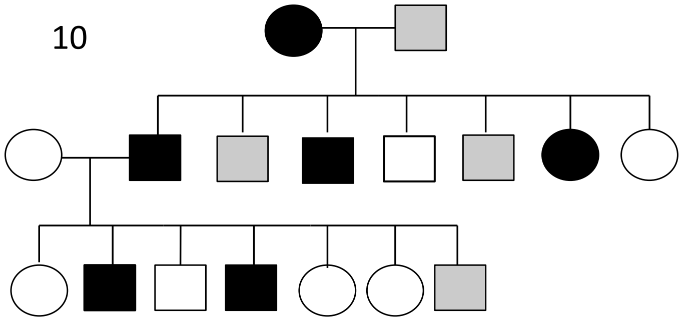

11

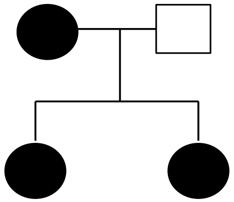

12

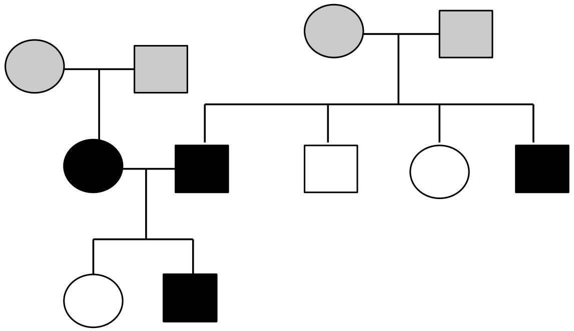

13

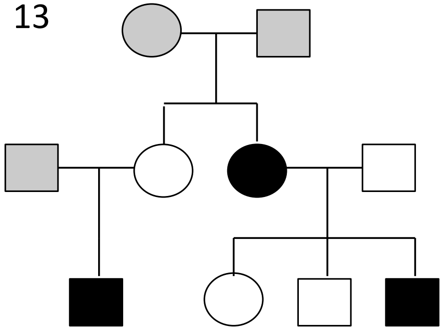

14

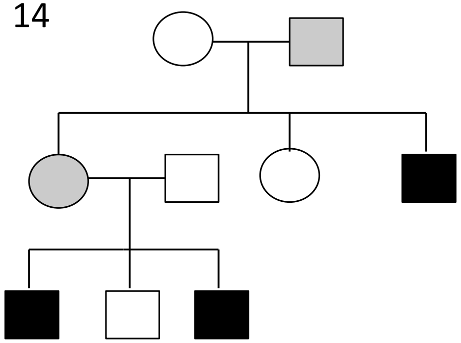

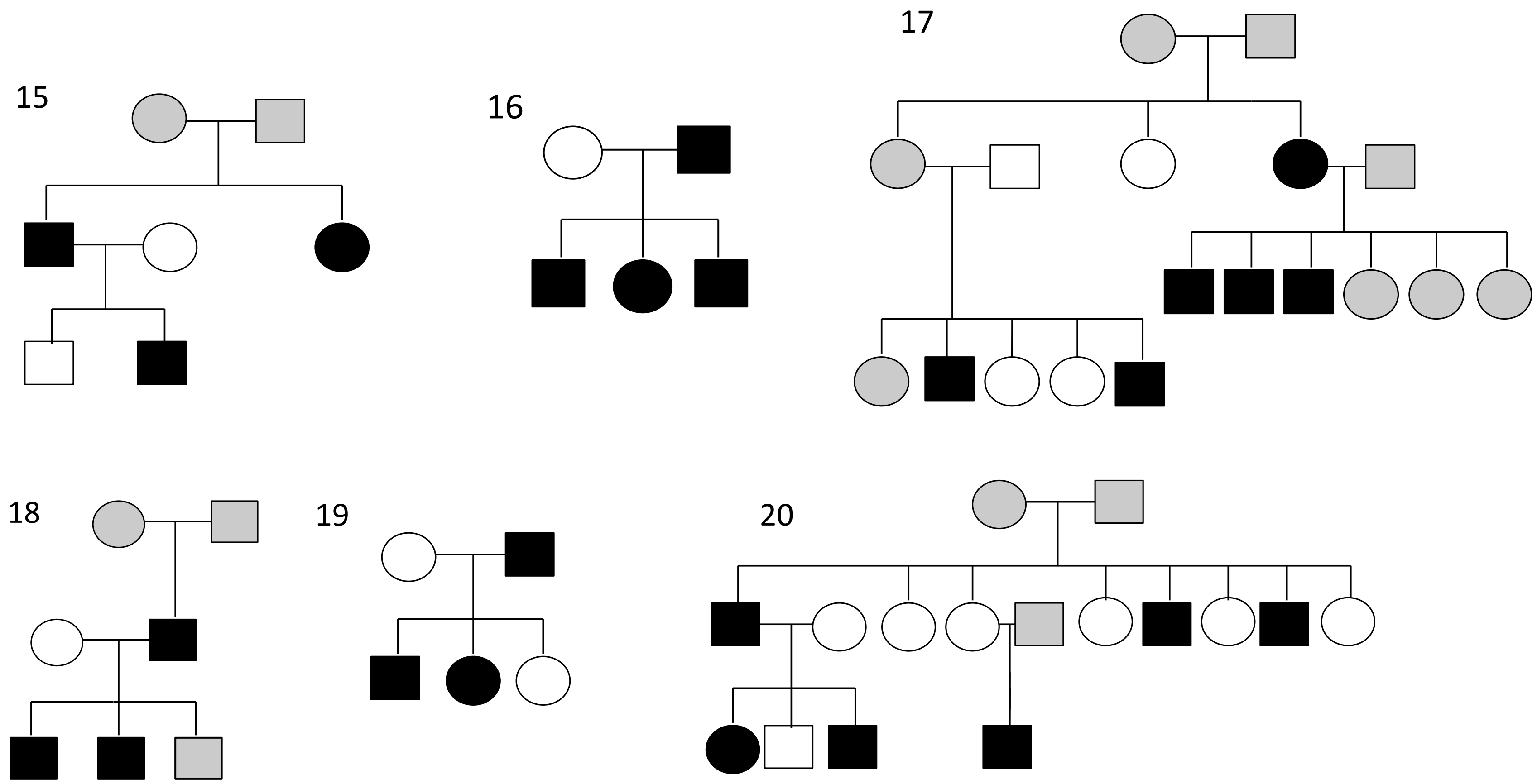


**Supplementary Figure 1** – 20 example cohort pedigrees from which probands were sent for initial whole exome sequencing

| species | match | gene | aa | alignment |
|---|---|---|---|---|
| Human | | | 375 | Q L E R R E Q R L R S R E E R L L H R A K E A L |
| mutated | not conserved | | 375 | Q L E R R E Q R L R S H E E R L L H R A K E A |
| Ptroglodytes | all identical | ENSPTRG00000012440 | 375 | Q L E R R E Q R L R S R E E R L L H R A K E A |
| Mmulatta | all identical | ENSMMUG00000030614 | 359 | Q L E R R E Q R L R S R E E |
| Fcatus | all identical | ENSFCAG00000012548 | 375 | Q L E R R E Q R L K S R E E R L L H R A K E A |
| Mmusculus | all identical | ENSMUSG00000045216 | 375 | Q L E R R E Q R L R N R E E R L L H R S K E A |
| Ggallus | all identical | ENSGALG00000002155 | 346 | Q L E R M E Q R I K N R E E R L L H R S N E A |
| Trubripes | not conserved | ENSTRUG00000015423 | 383 | Q Q E R R Q Q R I K N H I R Q S L T S K L E D |
| Drerio | no alignment | ENSDARG00000054754 | n/a | |
| Dmelanogaster | no alignment | FBgn0038755 | n/a | |
| Celegans | no alignment | Y34B4A.3 | n/a | |
| Xtropicalis | all identical | ENSXETG00000016769 | 370 | M E Q R K K N R E E R L L H R S N E A |

**Supplementary Figure 2 Multiple sequence alignment (m.s.a.) for the p.Arg375 (R375) residue.**

M.s.a. was generated using MutationTaster [5]. The p.Arg375 residue is highly conserved amongst different species, GERP score 4.3.

**References**


1. Pollard KS, Hubisz MJ, Rosenbloom KR, Siepel A. Detection of nonneutral substitution rates on mammalian phylogenies. Genome research 2010;20:110-21.
2. Kumar P, Henikoff S, Ng PC. Predicting the effects of coding non-synonymous variants on protein function using the SIFT algorithm. Nature protocols 2009;4:1073-81.
3. Adzhubei IA, Schmidt S, Peshkin L, et al. A method and server for predicting damaging missense mutations. Nature methods 2010;7:248-9.
4. Chun S, Fay JC. Identification of deleterious mutations within three human genomes. Genome research 2009;19:1553-61.
5. Schwarz JM, Cooper DN, Schuelke M, Seelow D. MutationTaster2: mutation prediction for the deep-sequencing age. Nature methods 2014;11:361-2.
6. Shihab HA, Gough J, Cooper DN, et al. Predicting the functional, molecular, and phenotypic consequences of amino acid substitutions using hidden Markov models. Human mutation 2013;34:57-65.
7. Kircher M, Witten DM, Jain P, O'Roak BJ, Cooper GM, Shendure J. A general framework for estimating the relative pathogenicity of human genetic variants. Nature genetics 2014;46:310-5.
8. Cooper GM, Stone EA, Asimenos G, et al. Distribution and intensity of constraint in mammalian genomic sequence. Genome research 2005;15:901-13.
